# Characterization of a new *Pm2* allele associated with broad-spectrum powdery mildew resistance in wheat line Subtil

**DOI:** 10.1038/s41598-017-18827-4

**Published:** 2018-01-11

**Authors:** Yuli Jin, Hongxing Xu, Pengtao Ma, Xiaoyi Fu, Liping Song, Yunfeng Xu, Xiaotian Zhang, Diaoguo An

**Affiliations:** 1 Center for Agricultural Resources Research, Institute of Genetics and Developmental Biology, Chinese Academy of Sciences, Shijiazhuang, Hebei, 050021 China; 2 Shijiazhuang Academy of Agricultural and Forestry Sciences, Shijiazhuang, Hebei, 050041 China; 3 The College of Life Science, University of Chinese Academy of Sciences, Beijing, 101408 China

## Abstract

Wheat powdery mildew is a severe disease affecting yield and quality. Host resistance was proved to be effective and environment-friendly. Wheat line Subtil is an elite germplasm resource resistant to 28 of 30 tested *Bgt* isolates. Genetic analysis showed that the powdery mildew resistance in Subtil was conferred by a single dominant gene, temporarily designated *PmSub*. Using bulked segregant analysis, *PmSub* was mapped to chromosome arm 5DS, and flanked by the markers *Bwm16* and *Cfd81/Bwm21* at 5.0 and 0.9 cM, respectively. Allelism tests further confirmed *PmSub* was allelic with documented *Pm2* alleles. Then, homologous sequences of *Pm2a* related sequence was cloned from Subtil and Chinese Spring. It was completely identical to the reported *Pm2a* sequence, but significantly different from that of Chinese Spring. A marker *SWGI067* was developed based on the sequence divergence of homologous sequence in Subtil and Chinese Spring. *SWGI067* was closely linked to *PmSub*, indicating that the gene *PmSub* itself was different from the cloned *Pm2a* related sequence. Meanwhile, Subtil produced significantly different reaction pattern compared with other genotypes with *Pm* genes at or near *Pm2* locus. Therefore, *PmSub* was most likely a new allele of *Pm2*. *PmSub* has opportunities for marker-assisted selecting for high-efficiency wheat improvement.

## Introduction

Wheat (*Triticum aestivum* L.) powdery mildew, caused by *Blumeria graminis* f. sp. *tritici* (*Bgt*), is one of the most damaging foliar diseases that occurs worldwide, especially with the deployment of dwarf and semi-dwarf cultivars and improvement of irrigation conditions^1–3^. Host resistance is proved to be an effective and safe method to minimize grain losses caused by the disease. However, resistance is often defeated by virulent mutants of the pathogen after long-term popularization of the cultivars with resistant gene(s)^4,5^. Previous studies indicated that most current wheat cultivars and breeding lines grown in China lacked effective resistance to powdery mildew (*Pm*)^6^. Therefore, it is urgent to identify more effective resistant sources among various germplasms to increase the genetic diversity of the resistant genes.

Up to now, 77 formally (*Pm1-Pm54, Pm8* is allelic to *Pm17, Pm18* = *Pm1c, Pm22* = *Pm1e, Pm23* = *Pm4c, Pm31* = *Pm21*) and more than 30 temporarily designated (e.g. *PmYB*, *PmWFJ*, *MlIw170*) wheat *Pm* genes have been reported at 56 loci throughout all homoeologous chromosome groups^7,8^. Among these genes, there is an interesting multi-allelic phenomenon, that is, several *Pm* genes with different reaction patterns to *Bgt* isolates were located at the same locus in different genotypes. These loci include *Pm1* (*Pm1a-1e*), *Pm2* (*2a-2c*), *Pm3* (*3a-3j*), *Pm4* (*4a-4d*), *Pm5* (*5a-5e*) and *Pm24* (*24a-24b*) that were located at 7AL, 5DS, 1AS, 2AL, 7BL and 1DS, respectively^7,9^. This could due to the plant-pathogen interaction during long term deployment of the resistant cultivars or multiple generations of hybridization^10,11^. The new alleles may be very useful evolution, because when some alleles have lost effectiveness, new allelic variation may be present in other materials and provide broader resistant spectrum to different *Bgt* isolates, such as *Pm2*, *Pm4* and *Pm5* in several Chinese cultivars which increased the diversity of available resistance genes^9,12,13^.

Molecular markers are powerful tools for tagging genes and markerassisted selection (MAS)^14^. Using various kinds of markers, many favorable genes have been mapped to specific chromosomal loci^7^. In particular, with the development of high-throughput single nucleotide polymorphism (SNP) genotyping platforms based on wheat 9 K, 90 K and even 660 K SNP chips, high density linkage maps can be conducted using the SNP markers which can greatly increase the number of markers closely linked to targeted genes^15–18^. Using closely linked markers, the valuable genes can be rapidly transferred to other cultivars or pyramided with other desirable genes. For example, three QTLs conferring powdery mildew resistance were effectively selected in both greenhouse and field experiments^19–21^, and this increased the powdery mildew resistance in pyramided lines. We previously reported that the gene *Pm2b* was transferred to various susceptible cultivars, such as Shimai 15, Shixin 828, Gao 8901 etc., and efficiently selected by its closely linked markers to improve the powdery mildew resistance of the susceptible cultivars^22^. Apart from disease resistance, QTL/genes for some major economic traits, such as grain protein content and pre-harvest sprouting tolerance, have also been used for MAS in wheat breeding programs^23–26^.

In this study, the wheat line Subtil is highly resistant to 30 of *Bgt* isolates from different regions of China at the seedling stage in the greenhouse and immune to *Bgt* composite mixture at the adult stage in the filed of Shijiazhuang city of China. To make better use of this resistance resource, the following studies were carried out to: (1) determine the inheritance of powdery mildew resistance in Subtil; (2) map the resistance gene(s) in Subtil, and confirm the allelic relationship with the documented *Pm* genes; (3) compare reaction patterns to different *Bgt* isolates between Subtil and the genotypes carrying documented *Pm* genes; (4) distinguish *PmSub* with the cloned *Pm2* sequence; and (5) investigate the applicability of closely linked markers for MAS.

## Results

### Inheritance of the powdery mildew resistance in Subtil

When inoculated with *Bgt* isolate E09, Subtil was immune with infection type (IT) 0, while Hengguan 35 was highly susceptible with IT 4. All the 25 F_1_ plants of Subtil × Hengguan 35 were immune with IT 0, in accord with that of the resistant parent, indicating the resistance gene in Subtil was dominant. Among the F_2_ population containing 162 plants, 119 were resistant with ITs 0–2; 43 were susceptible with ITs 3–4, fitting a single dominant gene segregation ratio (χ^2^_3:1_ = 0.13, *P* = 0.72) (Table 1). The F_2_ population was then transplanted to the field, and 141 plants survived to produce F_3_ seeds. When tested with the same isolate, the F_2:3_ families segregated as 43 homozygous resistant (RR), 64 heterozygous resistant (Rr) and 34 were homozygous susceptible (rr), which confirmed single gene segregation ratio (χ^2^_1:2:1_ = 2.35, *P* = 0.31). This gene was temporarily designated *PmSub*.

**Table 1:** Phenotype reactions of the F_2_ populations from the cross between Subtil and the documented resistance stocks with *Pm2a*, *Pm2b*, *Pm2c*, *PmLX66* and *Pm48* to the *Blumeria graminis tritici* (*Bgt*) isolate E09.

Crosses	Number of Resistant plants	Number of Susceptible plants
Subtil(*PmSub*) × Ulka/8*Cc (*Pm2a*)	1307	0
Ulka/8*Cc (*Pm2a*) × Subtil(*PmSub*)	1165	0
Subtil(*PmSub*) × KM2939 (*Pm2b*)	1490	0
KM2939 (*Pm2b*) × Subtil(*PmSub*)	1381	0
Subtil(*PmSub*) × Niaomai(*Pm2c*)	1932	0
Niaomai(*Pm2c*) × Subtil(*PmSub*)	226	0
Subtil(*PmSub*) × Liangxing66(*PmLX66*)	1476	0
Liangxing66(*PmLX66*) × Subtil(*PmSub*)	1341	0
Tabasco(*Pm48*) × Subtil(*PmSub*)	1405	1

### Molecular mapping of *PmSub*

**Figure 1:**
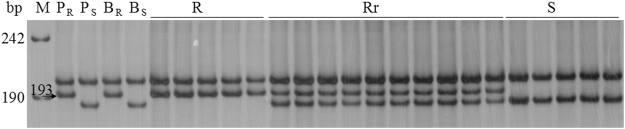
Examples of amplification patterns by the marker *Bwm20* from selected F_2:3_ of Subtil × Hengguan 35. M: pUC19/MspI; P_R:_ Subtil; P_S_: Hengguan 35; B_R_: Resistance bulked pool; B_S_: Susceptible bulked pool; R: Homozygous resistant F_2:3_ families; Rr: Heterozygous resistant F_2:3_ families; S: Homozygous susceptible F_2:3_ families. Black arrows indicated the polymorphic band (s).

**Figure 2:**
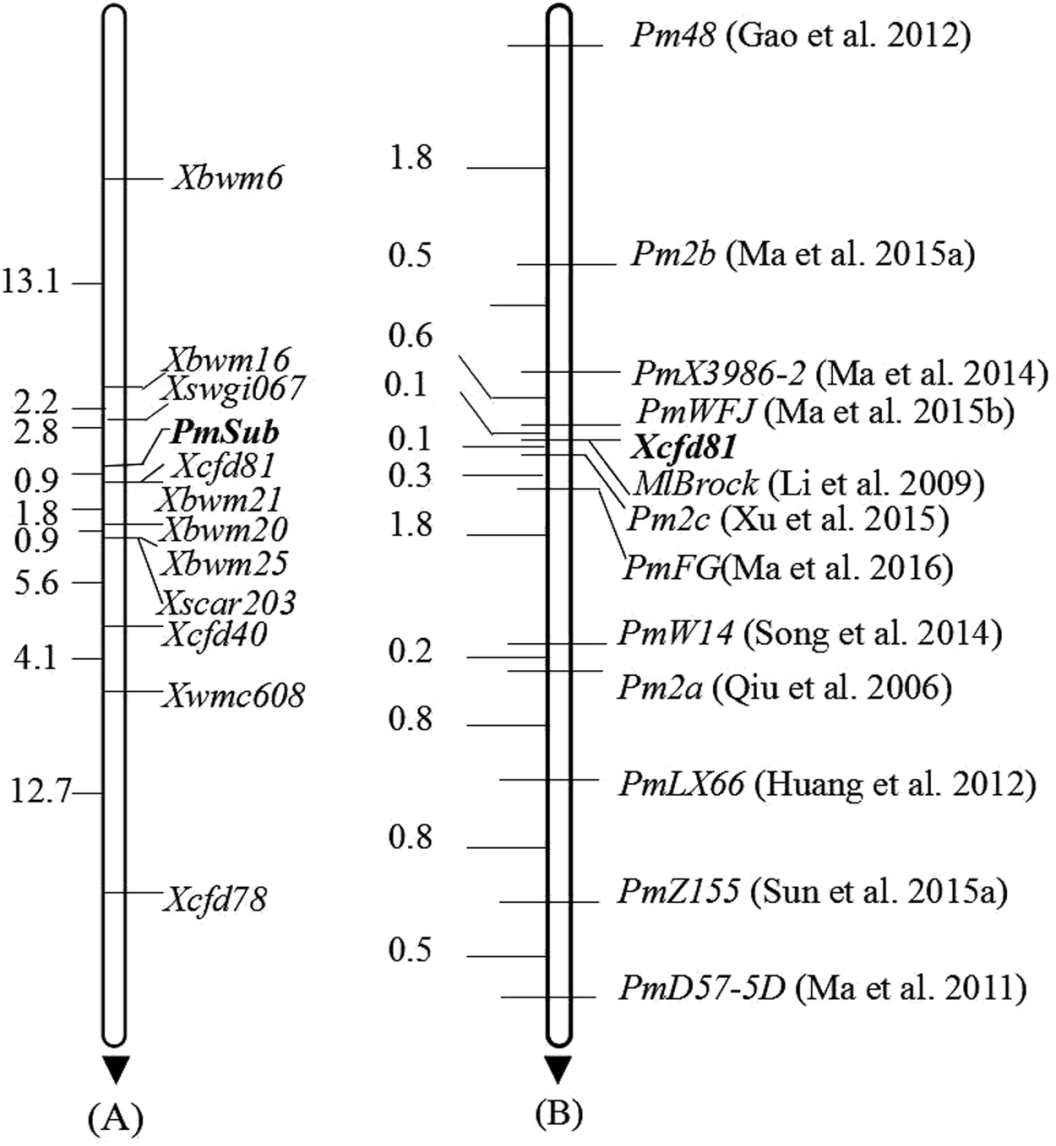
Linkage map of *PmSub* after genotyping on F_2:3_ families of Subtil × Hengguan 35 (**A**) and the comparison of loci between *PmSub* and part of the documented *Pm* genes at or near *Pm2* locus using the anchoring marker *Cfd81* (**B**). Genetic distances in cM are showed to the left.

Initially, 310 SSR markers were surveyed their polymorphisms between parents Subtil and Hengguan 35, and the resistant and susceptible DNA bulks. Only the marker *Cfd81* showed consistent polymorphism between the parents and bulks. Because *Cfd81* was tightly linked to *Pm2*^27^ and *Pm48*^28^, further 17 markers linked to *Pm2* alleles or *Pm48* were tested to survey the polymorphism between parents and bulks, including two SCAR markers *Scar112* and *Scar203*, five SSR markers *Gwm159*, *Cfd78*, *Wmc608*, *Cfd40* and *Wmc805* and 10 SNP-derived SSR markers *Bwm13*, *Bwm6*, *Bwm3*, *Bwm11*, *Bwm8*, *Bwm9*, *Bwm16*, *Bwm20*, *Bwm21* and *Bwm25*. Of these markers, 12 markers showed polymorphism between the parents and the bulks except for markers *Bwm3*, *Bwm8*, *Bwm9*, *Bwm11* and *Bwm13*. Then all the 13 polymorphic markers containing *Cfd81* were genotyped on the 141 F_2:3_ families of Subtil × Hengguan 35 (Fig. 1 & Fig. S1). Linkage analysis indicated that three markers *Scar112*, *Gwm159* and *Wmc805* were not linked to *PmSub*. A linkage map of *PmSub* was then constructed using the linked markers (Fig. 2). *PmSub* was flanked by the markers *Cfd81*/*Bwm21* (distal) and *Bwm16* (proximal) with genetic distances of 0.9 and 5.0 cM, respectively.

### The reaction patterns of Subtil and the lines with reported resistance alleles at or near the *Pm2* locus

**Figure 3:**
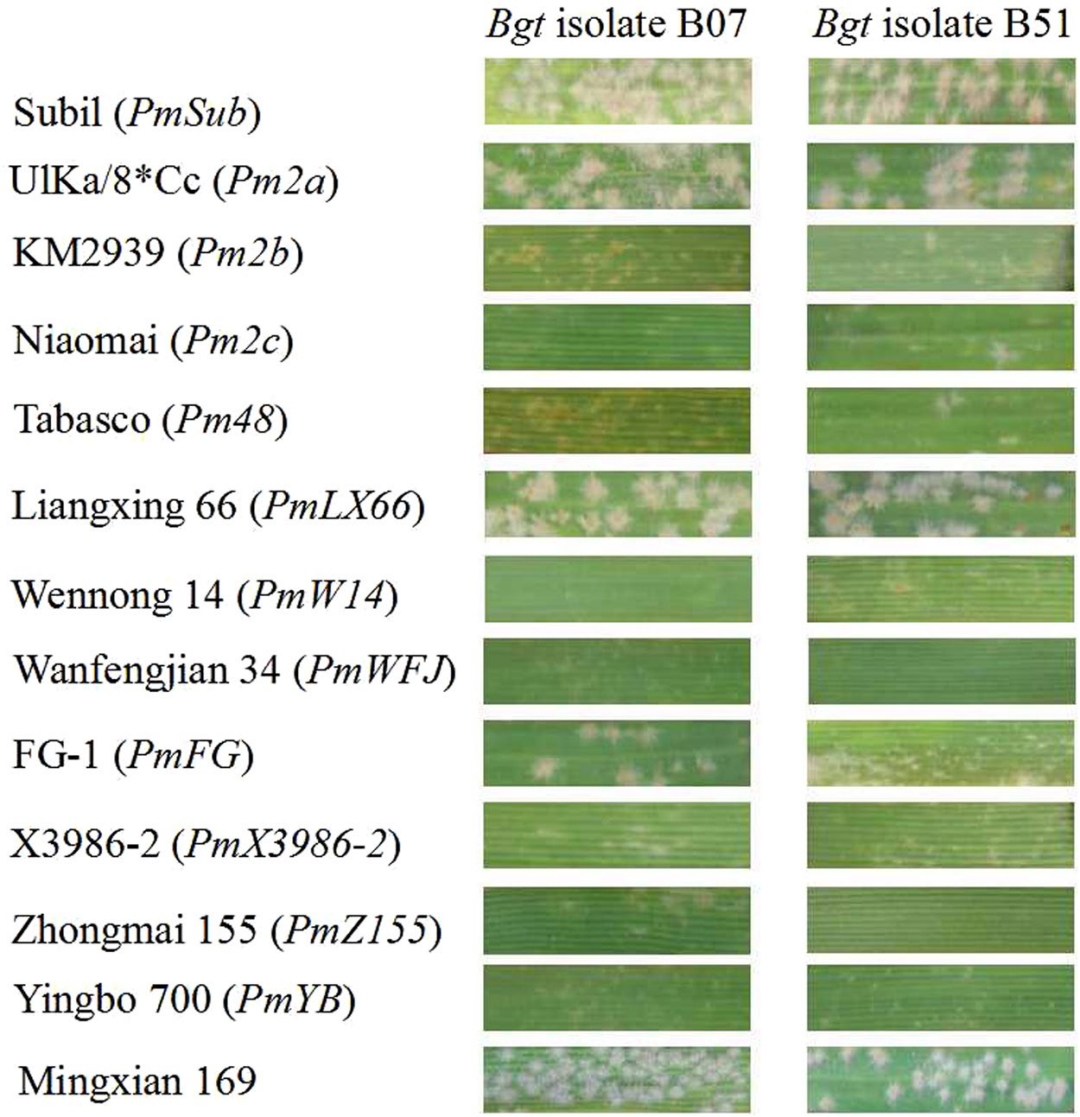
Examples of leaf segment reactions of Subtil and various wheat genotypes to 2 of 30 *Bgt* isolates; Mingxian 169 was used as the susceptible control.

When inoculated with 30 *Bgt* isolates, Subtil was resistant to 28 of 30 isolates, while Ulka/*8 Cc (*Pm2a*), KM2939 (*Pm2b*), Niaomai (*Pm2c*), Tabasco (*Pm48*), D57-5D (*PmD57-5D*), Liangxing 66 (*PmLX66*), X3986-2 (*PmX3986-2*), Wanfengjian 34, Yingbo 700, Wennong 14, Zhongmai 155 and FG-1 were susceptible to six, three, three, four, six, seven, eleven, seven, one, seven, seven and six of the tested isolates, respectively (Table 1 & Fig. 3). Subtil showed a relatively broader resistant spectrum to different *Bgt* isolates, and its reaction pattern was different from those of the documented resistant stocks with *Pm* genes at/near the *Pm2* locus. Thus, *PmSub* is most likely a new *Pm* gene.

### Allelism of *PmSub* and the documented *Pm* genes on chromosome 5DS

To identify the allelic relationship between *PmSub* and the documented *Pm* genes on chromosome arm 5DS, F_2_ populations of the reciprocal crosses between Subtil (*PmSub*) and Ulka/*8 Cc (*Pm2a*), KM2939 (*Pm2b*), Niaomai (*Pm2c*), Liangxing 66 (*PmLX66*) were tested against *Bgt* isolate E09 avirulent to all these resistant stocks. All tested F_2_ plants of Subtil × genotypes with *Pm2* alleles, including 10,318 plants of eight crosses, showed resistant to E09 (Table 2). Considering no susceptible plants were detected in these F_2_ populations, no recombination occurred between *PmSub* and *Pm2* allelic loci. This indicated that *PmSub* was allelic with *Pm2*. Combined with the distinguishable reaction pattern to the different *Bgt* isolates, *PmSub* is most likely a new allele at the *Pm2* locus. Furthermore, one of 1406 F_2_ plants of Subtil (*PmSub*) × Tabasco (*Pm48*) was susceptible to E09 with IT 4. This indicates that *PmSub* seems to be closely linked with *Pm48*.

**Table 2:** Reaction patterns of genotypes carrying documented *Pm* genes at or near *Pm2* locus after inoculating 30 *Blumeria graminis tritici* (*Bgt*) isolates.

Cultivar/lines	*Pm* gene	*Blumeria graminis tritici* isolates (*Bgt*^a^)																													
		E 01	E 02	E 03	E 05	E 06	E 07	E 09	E 11	E 13	E 16	E 17	E 18	E 20	E 21	E 22	E 23-1	E 23-2	E 26	E 30-1	E 30-2	E 31	E 32	E 49	E 50	B 07	B 13	B 14	B 41	B 45	B 51
Mingxian169	—	4^c^	4	4	4	4	4	4	4	4	4	4	4	4	4	4	4	4	4	4	4	4	4	4	4	4	4	4	4	4	4
Subtil	*PmSub*	0;	0	0	0;	0	0	0	0	0	0	0	0	1	0;	0	0	0	0	0	0	0	1	0	0	4	0	0	0	0	4
Ulka/8*Cc	*Pm2a*	0;	0;	0	4	0;	0;	0;	0;	0;	1	0;	4	4	0;	0	0;	0;	0;	0;	4	0	4	0;	0;	4	0	4	1	0;	4
KM2939	*Pm2b*	0;	0;	0;	0;	0;	0;	0;	0;	0;	0;	0;	1	2	3	0	0;	0;	0;	0;	0;	0;	3	0;	0;	1	0	4	0;	0;	0;
Niaomai	*Pm2c*	0;	0;	0	0	0	0;	0;	0;	0	0;	0;	0	0	0;	0;	0;	0;	0;	0;	0;	0;	4	0;	0;	0;	0;	0;	0;	0;	2
D57-5D	*PmD57-5D*	0;	0;	0;	4	0;	0;	0	0;	0;	0;	0;	3	4	0	0	0;	0;	0;	0;	4	0;	4	0	0	2	0	4	0;	0;	2
LiangXing66	*PmLX66*	0	0;	0	3	0;	0;	1	0;	0;	3	0;	4	4	0;	0	0;	1	0;	0;	4	0	4	0	0;	4	0	4	0;	0;	4
YingBo700	*PmYB700*	0	0	0	1	0	0	0	0	0	0	4	0	1	0	0	0	0	0	0	0	0	0	0	0	0	0	0	0	0	1
Wanfengjian 34	*PmWFJ*	—^b^	—	—	4	1	1	0	0;	—	0	4	4	0	0	2	4	0	—	—	0	0	4	4	4	0	—	2	0	—	0
Wennong 14	*PmW14*	—	—	—	3	0	0	0	0;	—	0	0	3	0	0	0	0	0	—	—	0	0	4	0	2	2	—	0	0	—	2
Zhongmai 155	*PmZ155*	—	—	—	3	0	0	0	0;	—	0	0	3	0	0	0	0	0	—	—	0	0	4	0	1	1	—	0	0	—	0
FG–1	*PmFG*	—	—	—	4	—	0	0	0	—	0	4	3	0	0	0	4	0	—	—	0	0	2	0	4	3	—	—	0	—	3
X3986–2	*PmX3986*–*2*	0;	4	0	4	0	0;	4	0;	0;	0;	0;	2	3	0;	4	0;	0;	0;	0;	4	3	4	0;	0;	1	4	4	4	0;	1
Tabasco	*Pm48*	0;	0;	0	3	0	0;	0	0	0;	0	0;	3	4	0	0	0	1	0;	0	0;	0;	4	0;	0;	0;	0	0	0;	0	2

^a^Each *Bgt* isolate represents a different race (Zhou *et al*. 2002).

^b^“–” represents no data.

^c^Infection types (IT) were scored on a 0–4 scale, where 0, 0;, 1 and 2 were considered as resistant and 3 and 4 as susceptible.

### Comparison of *PmSub* and the cloned *Pm2* sequence

Using primers JS320 and JS305, a band was produced from PCR, and it was 4825 bp after Sanger sequencing. Then, using the 4825 bp DNA as template, the nested PCR was performed to obtain the first exon, which was a 3730 bp sequence in the amplified band (4083 bp) after Sanger sequencing. Meanwhile, a significant sequence difference was present in Chinese Spring using the same procedure, including a 12 bp insertion, a 13 bp deletion, a 33 bp deletion and many single nucleotide mutations. Compared with the cloned *Pm2a*, the first exon was completely same with that of *Pm2a*. The second and third exons were amplified by the primers JS350 and JS313. After Sanger sequencing, they were 58 and 46 bp bands respectively and also completely same with the second and third exons of *Pm2a*. Therefore, the cloned exons from Subtil were identical with those of *Pm2a* and significantly different from that of Chinese Spring.

To confirm the cloned sequence from Subtil was the gene *PmSub* itself that is indeed correlated with the powdery mildew resistance in Subtil, marker *SWGI067* that can amplify part of the first exon was first used to test Subtil, Hengguan 35 and the resistant and susceptible bulks. It can amplify consistent polymorphism between parents and bulks. Then, *SWGI067* was used to genotyped on the 141 F_2:3_ families of Subtil × Hengguan 35. Seven recombinants were detected in the F_2:3_ families, among which the genotypes of four segregated F_2:3_ families (No. 51, 54, 96 and 139) were homozygous susceptible as Hengguan 35, two segregated F_2:3_ families (No. 63 and 130) were homozygous resistant as Subtil and one homozygous resistant F_2:3_ family (No. 141) was heterozygous resistant. To avoid false hybrid strains of these recombinants, 50 SSR markers randomly distributed on 21 chromosomes of wheat were used to detect their genetic backgrounds. The results showed that the genetic backgrounds of these recombinants were accorded with their parents, and the recombinations were really existing. This demonstated that the first exon of the *Pm2a* related gene had certain genetic distance with *PmSub*. After calculation by Mapmarker 3.0, the genetic distance was 2.8 cM (distal) (Fig. 1), indicating that *PmSub* was likely different from the cloned sequence of *Pm2a*.

### Potential of flanking markers for MAS

**Figure 4:**
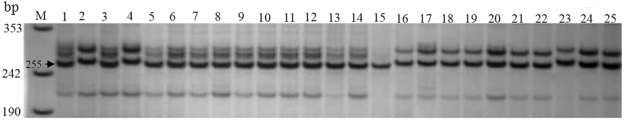
Amplification patterns of the marker *Cfd81* on Subtil, documented stocks with *Pm2* alleles and several wheat cultivars for validation of MAS. Lanes 1–25 are Subtil, Hengguan35, Resistance bulked pool, Susceptible bulked pool, Ulka/*8 Cc, KM2939, Niaomai, Brock, D57-5D, Liangxing 66, X3986-2, Zhongmai 155, Wennong 14, FG-1, Tabasco, Shi 4185, Shimai 15, Gao 8901, Han 6172, Han 7086, Kenong 9204, Jishi 02-1, Aikang 58, Baichun 5, Zhengmai 9023; M: pUC19/*MspI*; Black arrow indicates the polymorphic band in Subtil.

The flanking markers *Cfd81* and *Bwm16* of *PmSub* were assayed on 12 documented resistant stocks and 10 Chinese elite cultivars to investigate the potential of the markers for MAS. The polymorphic *Cfd81* and *Bwm16* alleles were present in Subtil and other *Pm2* stocks, whereas not appeared in the 10 cultivars, indicating that when *PmSub* was transferred to these cultivars with no *Pm* genes at the *Pm2* locus by conventional hybridization, the flanking markers *Cfd81* and *Bwm16* can be used in MAS (Fig. 4  & Fig. S2).

## Discussion

Subtil is a highly resistant wheat breeding line in China. In five consecutive years, Subtil was tested against different *Bgt* isolates at seedling stage in the greenhouse, and mixed *Bgt* isolates collected from different regions of wheat production at adult stage in the field. Subtil consistently showed a stable, high level of resistance to powdery mildew. To identify the powdery mildew resistance, the segregation population of Subtil × Hengguan 35 was constructed for genetic analysis and molecular mapping of the *Pm* gene (s) in Subtil. A single dominant gene on chromosome arm 5DS, temporarily designated *PmSub*, was proved to provide the powdery mildew resistance in Subtil.

On chromosome arm 5DS, a series of *Pm2* alleles (e.g. *Pm2a*, *Pm2b*, *Pm2c*, *PmD57-5D*, *PmLX66* and *PmX3986-2*, etc) and *Pm48* that closely linked to *Pm2* have been reported^22,28–34^. Compared with the previously reported genes, *PmSub* showed relatively broad resistant spectrum, and was significantly different from the documented *Pm* genes on chromosome arm 5DS. Furthermore, allelism tests of not only between *PmSub* and *Pm2* alleles but also *PmSub* and *Pm48* were carried out to thoroughly confirm the allelic relationship with the documented *Pm* genes.

Recently, the *Pm2a* related sequence was cloned by mutant chromosome sequencing^35^. The homologous sequence was cloned in Subtil in this study. Although the homologous sequence in Subtil was same as the cloned *Pm2a* related sequence, it was closely linked but not co-segregated with *PmSub*, indicating that the cloned homologous sequence may not be the *PmSub* itself but a key factor of gene, and also be different from the cloned *Pm2a* related sequence. We presume that the reasons may be as follows: the cloned *Pm2a* sequence may not be the *Pm2a* gene itself, but a key factor in the upstream region of *Pm2a*. When this sequence is mutated, it will also lose the function. Although the homologous sequence in Subtil is same as the cloned *Pm2a*, it doesn’t mean that the *PmSub* sequence was same as the *Pm2a* sequence. More work need to be done in the future. For example, functional complemention verification by transgenosis need to be done to confirm if the function was obtained by transferring the cloned sequence of *Pm2a*; map-based cloning should be performed sequentially using forward genetic approaches to confirm the gene itself, etc.

In this study, a new allele joined the complex *Pm2* allelic family. Like *Pm1*, *Pm3*, *Pm4* and *Pm5*, and more and more new alleles with different resistant spectrum to multiple *Bgt* isolates were identified in *Pm2* locus in recent years^8,22,31–34^. Previous studies indicated that part of the sequence variation may contribute to the phenomenon. For example, cloning of *Pm3b*^10,36^ demonstrated that about 3% sequence variation may result in different resistant spectrum of a series of *Pm3* alleles. In the past, identification of new resistant resources mainly focus on the genes located at new loci^7^. With more and more resistance genes have been identified, exploring new alleles of the documented genes was also important for increasing the genetic diversity of the resistance genes. Even if the documented resistance genes in the commercial cultivars have lost or reduced resistance, their new allelic variations may have broader resistant spectrum to the virulent isolates, such as many new alleles of *Pm2*, *Pm4* and *Pm5*^12,13,22,33,34,37^. These allelic variations can increase the diversity at this locus, which will contribute to not only the genetic improvement of crops, but also the understanding of mechanism in the host-pathogen interactions^38,39^.

In wheat breeding, MAS is a rapid and effective way to transfer or pyramid excellent traits compared with conventional breeding methods. It has been used in many traits improvement successfully, such as disease resistance, adverse element tolerance and quality traits, etc^40^. In MAS, the key factor is the selection of applicable molecular markers^41^. In this study, Subtil is a wheat breeding line with superior agronomic performance. So, when the broad spectrum resistance gene *PmSub* was identified in Subtil, potential of flanking markers for MAS was investigated. Fortunately, two flanking markers have potential to detect *PmSub* in the tested cultivars with no *Pm* genes at or near *Pm2* locus. Therefore, Subtil can be crossed to these cultivars, and their progenies can be high-efficiently selected by *Cfd81* and *Bwm16* for resistance breeding. Meanwhile, one other thing to note is the selecting marker *Bwm16*. Unlike *Cfd81*, it has a relatively far distance to *PmSub*, which will affect the efficiency and accuracy for MAS. So, more closely linked markers should be developed for MAS. Fine mapping and map-based cloning of *PmSub* will facilitate usefulness of this gene in wheat improvement.

## Materials and Methods

### Plant materials and *Bgt* isolates

Subtil is a winter wheat breeding line that is highly resistant to powdery mildew at both seedling and adult stages. Wheat cultivar Hengguan 35 is highly susceptible to powdery mildew and hence was used as susceptible parent in this study. An F_2_ population and 141 F_2:3_ families from the cross Subtil × Hengguan 35 were used to study the inheritance of powdery mildew resistance and map the resistance gene (s) in Subtil. The resistant stocks KM2939 (*Pm2b*)^22^, Niaomai (*Pm2c*)^8^, LiangXing 66 (*PmLX66*)^31^, X3986-2 (*Pm3986-2*)^32^, Wanfengjian 34 (*PmWFJ*)^33^; Yingbo 700 (*PmYB*)^34^, Wennong 14 (*PmW14*)^42^, Zhongmai 155 (*PmZ155*)^3^ and FG-1 (*PmFG*) are preserved in our lab. The resistant stock Ulka/*8 Cc (*Pm2a*)^29^, D57-5D (*PmD57-5D*)^30^ and German cultivar Tabasco (*Pm 48*)^28^ were provided by Prof. Hongyan Liu, Institute of Plant Protection, Henan Academy of Agricultural Sciences, Zhengzhou, Prof. Zhengqiang Ma in the Applied Plant Genomics Laboratory of Nanjing Agricultural University, Nanjing, and Prof. Shibin Cai, Institute of Food Crops, Jiangsu Academy of Agricultural Science, Nanjing, respectively. These resistant stocks were used in multi-isolates response comparisons with Subtil. Ten wheat cultivars in China were tested using molecular markers closely linked to the *Pm* gene in Subtil to validate the applicability of markers for MAS. Susceptible wheat cultivar Mingxian 169 was used as susceptible control for the test of powdery mildew resistance. Wheat cultivar Chinese Spring was used as the negative control which didn’t carry a *Pm2* allele when homology-based cloning *Pm2* alleles.

Twenty-eight single-pustule-derived powdery mildew virulent isolates were used to inoculate Subtil and the documented resistant stocks to test their reaction pattern to these *Bgt* isolates. These *Bgt* isolates have different virulence, and were kindly provided by Prof. Yilin Zhou, the State Key Laboratory for Biology of Plant Disease and Insect Pests, Institute of Plant Protection, Chinese Academy of Agricultural Sciences, Beijing. *Bgt* isolate E09 prevalent in North China was used to inoculate the mapping population of Subtil × Hengguan 35 for genetic analysis.

### Phenotyping reactions to *Bgt* isolates

The reactions to *Bgt* isolates were tested in a greenhouse with a high humidity environment at 18 C/12 °C (day/ night) with a photoperiod of 12–14 h of light per day^43^. The Mingxian 169 seedlings were inoculated 30 *Bgt* isolates respectively and preserved separately in the glass tubes. The genotypes with documented *Pm* resistance alleles at/near *Pm2* locus were grown in 128-well (3 cm × 3 cm) rectangular trays. Mingxian 169 was planted randomly in the trays as a susceptible check. These rectangular trays were prepared 30 copies. When the seedlings grown to one-leaf stage, every copy was inoculated a single *Bgt* isolate and preserved separately in a space. The F_2_ and F_2:3_ plants derived from the cross Subtil × Hengguan 35. *Bgt* isolate E09, avirulent on Subtil and virulent on Hengguan 35, was selected to inoculate Subtil, Hengguan 35 and their derived F_1_ hybrids, F_2_ and F_2:3_ populations (24 seedlings per family) of Subtil × Hengguan 35 for phenotypic survey and genetic analysis of the mapping populations. When the pustules were fully developed on the first leaf of Mingxian 169 at about 14–15 days after inoculation, infection types (ITs) for each plant were assessed on a 0–4 scale, and plants with ITs 0–2 were regarded as resistant and those with ITs 3 and 4 susceptible^43^.

### Genotyping of the mapping population and map construction

Total genomic DNA of Subtil, Hengguan 35 and their derived F_2:3_ familes were separated from their young seedling leaves following the procedure of Ma *et al*.^44^. Resistant and susceptible DNA bulks were produced by mixing equal amounts of DNA of 10 homozygous resistant and 10 susceptible F_2:3_ families of Subtil × Hengguan 35 for bulked segregant analysis (BSA)^45^.

SSR markers evenly distributed across all 21 wheat chromosomes^14,46,47^ were selected to perform polymorphic marker survey between the parents and bulks. Sequence characterized amplified region (SCAR) markers *SCAR203* and *SCAR112*, and SSR markers *BWM3*, *BWM6*, B*WM8*, *BWM9*, *BWM11*, *BWM16*, *BWM20*, *BWM21* and *BWM25* developed by Li *et al*.^48^ and Lu *et al*.^49^ respectively were also used to increase the marker density at the targeted interval. PCR was performed in a reaction volume of 10 ul containing 10–20 ng of template DNA, 2 pmol of each of the primers, 2 nmol of the dNTPs, 15 nmol of MgCl_2,_ 0.1 U of Taq DNA polymerase, and 1 × PCR buffer. The PCR profile was one cycle of 94 °C for 3 min followed by 35 cycles of 94 °C for 30 s, 50–65 °C (depending on specific primers) for 40 s and 72 °C for 40 s, with a final extension at 72 °C for 5 min. PCR products were separated in 8% non-denaturing polyacrylamide gels (Acrylamide: Bisacrylamide = 25:1 or 39:1) with 1 × TBE buffer (90 mM tris-borate, 2 mM EDTA, PH 8.3), and visualized by silver straining^50^. Polymorphic markers were then genotyped on the F_2:3_ families of Subtil × Hengguan 35. After confirming the response genotypes through progeny testing of F_2:3_ families using the *Bgt* isolate E09, Chi-squared (χ^2^) tests were used to determine the goodness-of-fit of observed data with expected segregation ratios. Linkage analysis between polymorphic markers and the *Pm* gene in Subtil was performed by Mapmarker 3.0 with a LOD threshold score of 3.0^51^. Genetic distances were estimated from recombination values using the Kosambi mapping function^52^.

### Allelism test of the *Pm* gene (s) and the documented *Pm* genes on the same chromosome arm

After the *Pm* gene (s) in Subtil was mapped on chromosome arm 5DS, Subtil was crossed with the genotypes with documented *Pm* genes on the same chromosome arm, including Ulka/*8 Cc (*Pm2a*), KM2939 (*Pm2b*), Niaomai (*Pm2c*), Liangxing 66 (*PmLX66*) and Tabasco (*Pm48*). The F_2_ populations were inoculated with the *Bgt* isolate E09, which was avirulent to Subtil, Ulka/*8 Cc, KM2939, Niaomai, Liangxing 66 and Tabasco. From the ratio of resistant and susceptible numbers, the allelic relationships between the *Pm* gene(s) in Subtil and documented *Pm* genes were confirmed.

### Molecular and genetic comparison of the *Pm* gene in Subtil with the cloned *Pm2* sequence

Based on the recent report about cloning of a *Pm2* allele *Pm2a*^35^, homologous sequence of *PmSub* was cloned using homology-based cloning. The first exon was firstly amplified using primers JS320 (Forward 5′-3′: ACGATGATGTGAATCTTCCGTG) and JS305 (Reverse 5′-3′: AATGATAGCATGCATTTGGAG). On this basis, the nested PCR was carried on to obtain the final sequence of the first exon using primers JS314 (Forward 5′-3′: TTTTCGCGGTATTGCTGGTG) and JS315 (Reverse 5′-3′: ACCTCCTGTCATCGGTTCAC). The second and third exons were obtained by JS350 (Forward 5′-3′: CCCTCCTCCTTGAAGAATCTGA) and JS313 (Reverse 5′-3′: GCACAAACTCTACCCTGTTCC). Then, the sequence of the *PmSub* were assembled and compared with the cloned sequence of *Pm2a*.

Based on the sequence divergence of the first exon in Subtil and Chinese Spring, a pair of primer *SWGI067* was designed (Forward 5′-3′: CCTGGGAGGGCTCGGATCACTG, Reverse 5′-3′: GGAGGGATGAGCGGTTCTGTAG). The amplified sequence of *SWGI067* can cover the diversity sequence interval of the first exon. Then, *SWGI067* was used to genotype on the F_2:3_ familes of Subtil × Hengguan 35. If the cloned sequence of Subtil was indeed the gene *PmSub* itself, the marker *SWGI067* will be co-segregated with the phenotype of F_2:3_ familes of Subtil × Hengguan 35, and if not, the gene *PmSub* itself may not be and different from the cloned sequence of *Pm2a*.

### Validation of the closely linked markers in different genetic backgrounds

To evaluate the potential of the *Pm* gene(s) in Subtil for MAS, the flanking markers were tested against Subtil, 12 documented resistant stocks with *Pm2* alleles and 10 Chinese wheat cultivars susceptible to powdery mildew. The patterns of the polymorphic bands were compared to assess the applicability of the markers in MAS. If polymorphic alleles were amplified between these wheat cultivars and Subtil, the markers can be used to detect the *Pm* gene (s) in Subtil when it was transferred to those cultivars by hybridization.

## Electronic supplementary material

Supplementary Information
