# Supplementary material for: Characterization of a new Pm2 allele associated with broad-spectrum powdery mildew resistance in wheat line Subtil
Source: Sci Rep. 2018 Jan 11;8:475. doi: 10.1038/s41598-017-18827-4 (PMC5765050; doi:10.1038/s41598-017-18827-4)

Characterization of a new *Pm2* allele associated with broad-spectrum powdery mildew resistance in wheat line Subtil

Yuli Jin[1+], Hongxing Xu[1+], Pengtao Ma[1+], Xiaoyi Fu[2], Liping Song[1], Yunfeng Xu[1], Xiaotian Zhang[1] & Diaoguo An[1*]

[1]Center for Agricultural Resources Research, Institute of Genetics and Developmental Biology, Chinese Academy of Sciences, Shijiazhuang, Hebei 050021, China

[2]Shijiazhuang Academy of Agricultural and Forestry Sciences, Shijiazhuang, Hebei 050041, China

[+]These authors contributed equally to this work

Correspondence and requests for materials should be addressed to D.A. (email: dgan@sjziam.ac.cn)

Full-length gel of Figure 1

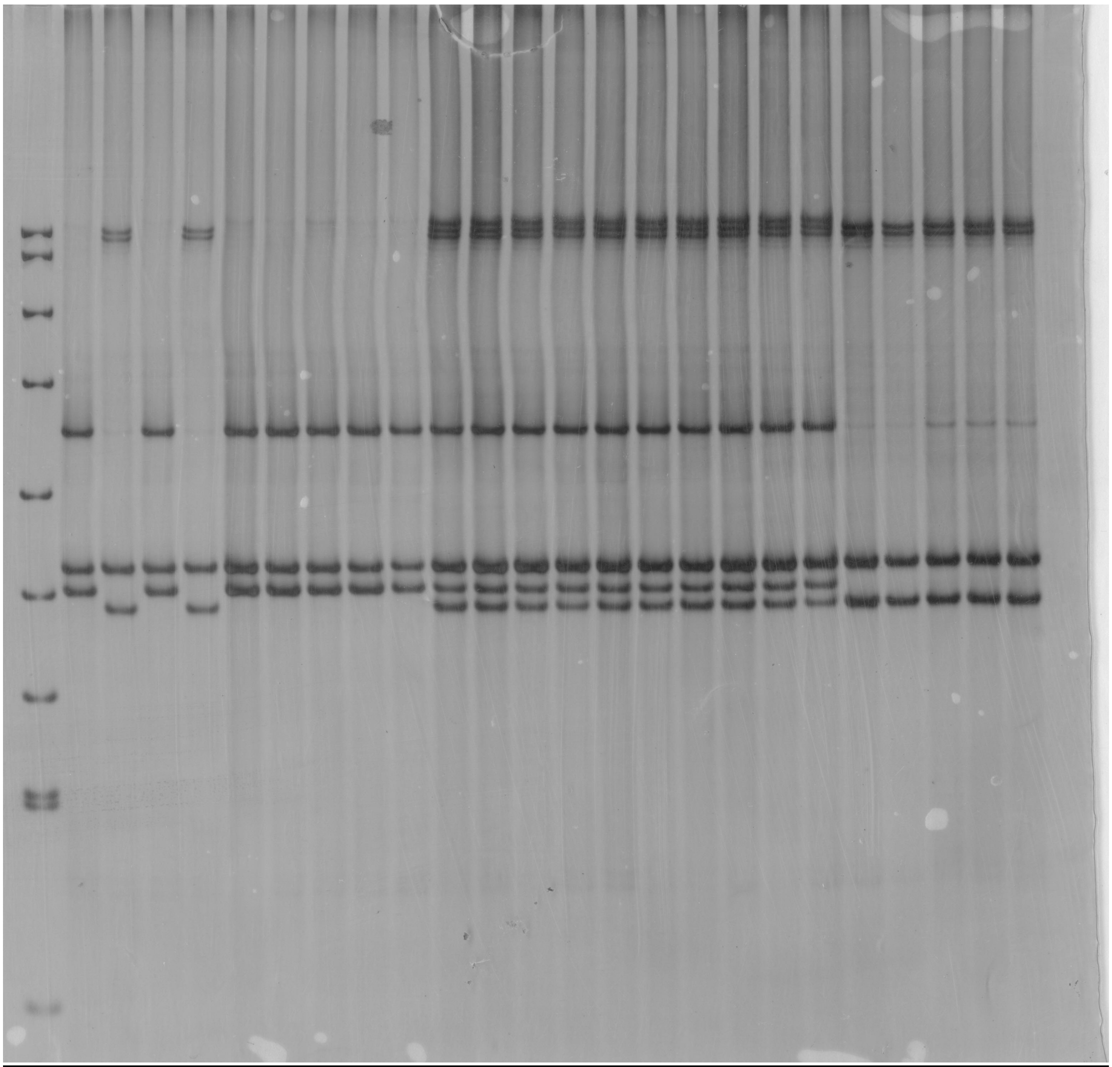

Full-length gel of Figure 3

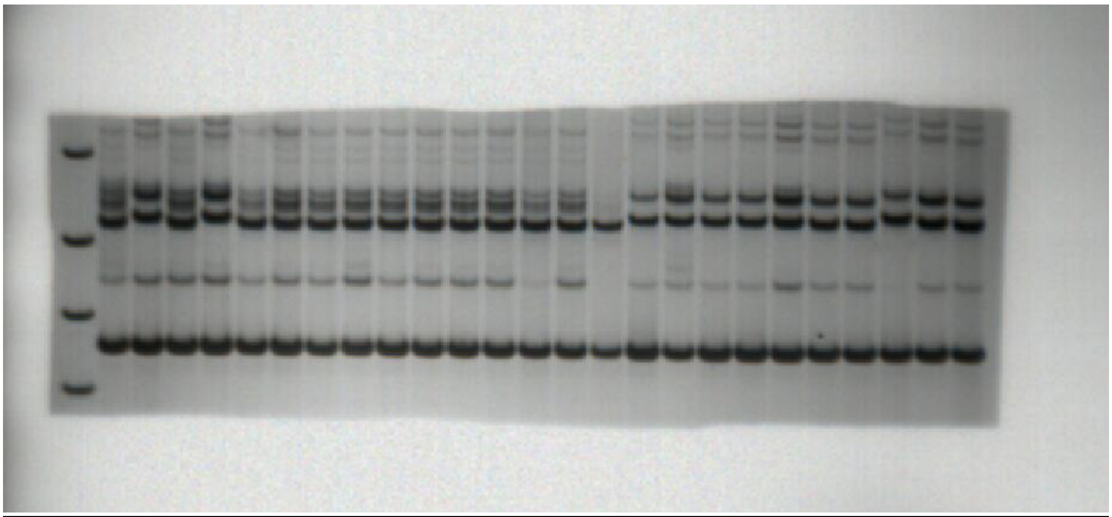

Supplement: Supplementary file 1 — Supplementary Information [file 41598_2017_18827_MOESM1_ESM.pdf]
